# *Escherichia coli* survival in response to ciprofloxacin antibiotic stress correlates with increased nucleoid length and effective misfolded protein management

**DOI:** 10.1098/rsos.230338

**Published:** 2023-08-09

**Authors:** George Butler, Julia Bos, Robert H. Austin, Sarah R. Amend, Kenneth J. Pienta

**Affiliations:** ^1^ Cancer Ecology Center, The Brady Urological Institute, Johns Hopkins School of Medicine, Baltimore, MD, USA; ^2^ Institut Pasteur, Université de Paris Cité, CNRS UMR 3525, Unité Plasticité du Génome Bactérien, Paris, France; ^3^ Department of Physics, Princeton University, Princeton, NJ, USA

**Keywords:** filamentation, evolution, resistance, antibiotics

## Abstract

The evolution of antibiotic resistance is a fundamental problem in disease management but is rarely quantified on a single-cell level owing to challenges associated with capturing the spatial and temporal variation across a population. To evaluate cell biological phenotypic responses, we tracked the single-cell dynamics of filamentous bacteria through time in response to ciprofloxacin antibiotic stress. We measured the degree of phenotypic variation in nucleoid length and the accumulation of protein damage under ciprofloxacin antibiotic and quantified the impact on bacterial survival. Increased survival was correlated with increased nucleoid length and the variation in this response was inversely correlated with antibiotic concentration. Survival time was also increased through clearance of misfolded proteins, an unexpected mechanism of stress relief deployed by the filamentous bacteria. Our results reveal a diverse range of survival tactics employed by bacteria in response to ciprofloxacin and suggest potential evolutionary routes to resistance.

## 1. Introduction

Antibiotic resistance is a major threat to global health, in 2019, over 1 million deaths were the result of resistant bacterial infections [1]. Antibiotic resistance is driven, in part, by de novo mutations [2] that confer phenotypic resistance in the form of upregulated efflux pumps, altered target protein binding, and improved enzymatic antibiotic degradation [3], among other mechanisms. Yet, while the molecular drivers of resistance are well known, the mechanisms by which these mutations arise (i.e. evolutionary kinetics) remain broadly unresolved [4], and can be explained by multiple non-mutually exclusive models.

A classical model of mutagenesis posits that mutations gradually accumulate over time within a population. Then, when a population is exposed to an antibiotic, the beneficial resistant mutations are selected for leading to the emergence of a resistant population [5]. A key concept in a classical model of mutagenesis is that mutations are already present within the population prior to the application of antibiotic stress. By contrast, a stress-induced model of mutagenesis argues that mutational rates can increase under periods of intense stress, which in turn increases the likelihood that a resistant phenotype will emerge [6]. The dynamic nature of stress-induced mutagenesis [7] is a departure from a classical model of mutagenesis because it means that a resistant mutant is not necessarily present within the population prior to the application of therapy. Distinguishing between these two models—selection versus adaptation is critical to understanding—and to addressing—the emergence of antibiotic resistance.

The first requirement of a resistant phenotype is survival under antibiotic stress. While antibiotic resistance mechanisms have been described at a molecular level [8], the temporal phenotypic dynamics of bacterial survival under stress at the single-cell level remain considerably less well defined [9]. Quantifying the phenotypic dynamics of bacteria under antibiotic stress is an important step to delineate the evolutionary mechanisms of resistance. In a classical model of mutagenesis in which a pre-existing mutation is selected for, phenotypic heterogeneity is expected to be limited and the resistant cellular genotype is expected to be similar to the starting population. By contrast, a stress-induced mechanism acts through a dynamic interplay between a cell and the stressor [7]. As a result, we expect that the phenotype of a surviving cell will also be dynamic. If true, then we would hypothesize that the degree of phenotypic variability in survival dynamics will be inversely correlated with antibiotic concentration. Hence, under higher concentrations of antibiotic that block the growth of the majority of susceptible bacteria, the variation in phenotypic dynamics strongly associated with survival will decrease as survival becomes increasingly more challenging (only the cells with higher fitness will survive) [10]. The difference in kinetics and timing between a classical and stress-induced model of mutagenesis is important because it suggests that mutagenic mechanisms could be a potential therapeutic target. Thus, treatment regimens could be developed to target treatment sensitive cells while also targeting mutagenic mechanisms to slow down the evolution of resistance [11].

**Figure 1.**
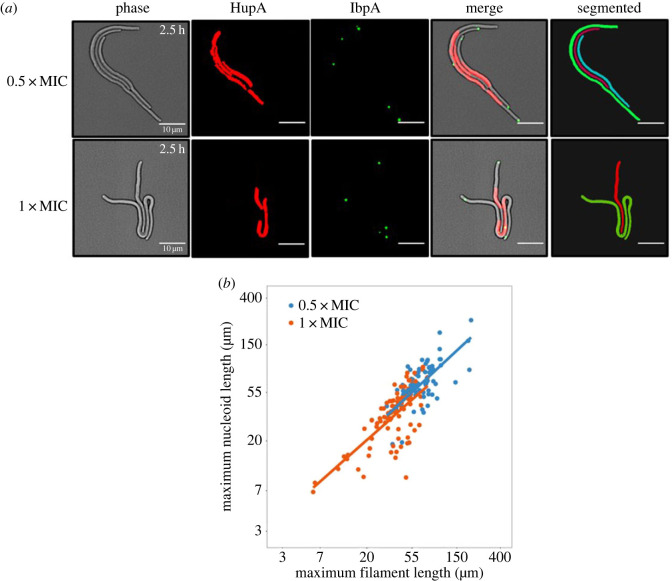
Measuring the response of ciprofloxacin antibiotic in filamentous *E. coli*. (*a*) Microscopy images of *E. coli* filamentation in response to 2.5 h of 0.5xMIC and 1xMIC of ciprofloxacin stress. From left to right: phase contrast image, fluorescent image (green channel) of misfolded protein aggregates targeted by the IbpA chaperone (IbpA-YFP), fluorescent image (red channel) of the nucleoid (histone-like protein (HU-mCherry)), merge image of phase contrast and two fluorescent channels, and segmented masks of filamentous cells showing the output of our segmentation algorithm. Two concentrations of ciprofloxacin are used: 0.5xMIC at which nucleoid length is typically spread along the filament body, and 1xMIC at which elongated filaments are seen with smaller nucleoid lengths that remain at the filament centre. Several discrete misfolded protein foci can be seen at the filament tips at both treatment levels. (*b*) A plot of the natural log-transformed maximum nucleoid length plotted against the natural log-transformed maximum filament length. The straight lines represent the fitted models for each treatment level. The maximum filament length is significantly positively correlated with the maximum nucleoid length at both the 0.5xMIC (*β* = 0.871, *p* < 0.1 × 10^−3^, *n* = 206) and the 1xMIC (*β* = 0.832, *p* < 0.1 × 10^−3^, *n* = 206) treatment levels.

To test these hypotheses and explore single-cell phenotypic responses to antibiotic treatment, we evaluated phenotypic variability in survival dynamics in *Escherichia coli* (*E. coli*), a model system for stress-induced mutagenesis [12], when exposed to various concentrations of ciprofloxacin antibiotic (sub-minimal inhibitory concentration (sub-MIC) and MIC). Ciprofloxacin is a fluoroquinolone antibiotic that shows increased rates of resistance in bacteria in recent decades [13–16]. Ciprofloxacin promotes a complex response leading to high levels of genetic and non-genetic heterogeneity [17]. Ciprofloxacin-induced genetic variation manifests via widespread DNA damage caused by DNA replication defects resulting in double-strand breaks [18] and increased mutational rates that trigger the SOS stress response [19,20]. Non-genetic ciprofloxacin-induced heritable variation arises owing to increased levels of reactivate oxygen species that damage the proteome through protein misfolding and a loss of function [21,22]. In turn, genetic and non-genetic mechanisms of proteome diversification drive high levels of phenotypic variability, potentially increasing the rate of adaptation and resistance [23–25]. Ciprofloxacin treatment leads to the formation of elongated cells or filamentous cells, mostly owing to the SOS-induction of changes in the functionality of sulA (formerly *sfiA* for suppressor of filamentation) (figure 1) [11,26–28] The induction of a filamentous morphotype is key because it serves as a marker for cells that are experiencing stress but remain metabolically active, a requirement for a stress-induced model of mutagenesis and in contrast to a non-growing persister subpopulation.

We used the filamentous phenotype and built a bespoke semi-automated image analysis pipeline to track *E. coli* cells under ciprofloxacin stress over time at a single-cell level. We also used a dual florescent reporter to simultaneously capture cell morphological characteristics, nucleoid dynamics, and misfolded protein aggregates. We found that filamentous cells with increased nucleoid length have an increased survival across treatment levels but that the variation in the response is inversely correlated with antibiotic concentration, highlighting a potential source of sub-MIC antibiotic evolvability. Using this unbiased longitudinal imaging approach, we also observed asymmetric tip-divisions as a means to clear misfolded protein aggregates. Finally, we demonstrate that a dynamic interplay of cellular responses emerges during cellular filamentation, but that the heterogeneity in response is more diverse at sub-MIC levels.

## 2. Results

### 2.1. Cell length and nucleoid dynamics at varying levels of ciprofloxacin stress

Ciprofloxacin is known to drive the formation of elongated filamentous *E. coli* cells with aberrant nucleoid dynamics (figure 1*a*). We treated a dual fluorescent reporter *E. coli* strain with IbpA-YFP and HupA-mCherry fluorescent reporters (see ‘Methods’ for detailed information) with 1xMIC or 0.5xMIC ciprofloxacin for 24 h. We evaluated the maximum filament length (from brightfield) and the maximum nucleoid length (HU-mCherry signal) as an estimate of DNA content. We found that there was significant variation among all groups in both mean maximum filament length (ANOVA; *p* < 0.1 × 10^−3^, *n* = 310; electronic supplementary material, figure S2*a*) and mean maximum nucleoid length (*p* < 0.1 × 10^−3^, *n* = 310; electronic supplementary material, figure S2*b*). Both ciprofloxacin treatment groups formed significantly longer filaments, and had significantly longer nucleoid length compared to control, though the 1xMIC treatment group had significantly shorter filaments and nucleoid length compared to the 0.5xMIC treatment group (*post hoc* Bonferroni multiple comparison test; electronic supplementary material, table S1).

Next, to investigate the relationship between cell and nucleoid elongation, we fitted a linear mixed model across the two ciprofloxacin treatment levels whereby the maximum nucleoid length is dependent on the maximum filament length. We set the treatment as a fixed effect and estimated a single intercept for two treatment levels but allowed the slopes to vary between treatment levels (see Methods for more details). We found that the maximum nucleoid length was positively correlated with the maximum filament length in both the 0.5xMIC (*β* = 0.871) and 1xMIC treatments (*β* = 0.832; figure 1*b*) and the model explained a significant proportion of the variation in the maximum nucleoid length (marginal *R*^2^ = 0.625).

### 2.2. Misfolded protein dynamics at varying levels of ciprofloxacin stress

**Figure 2.**
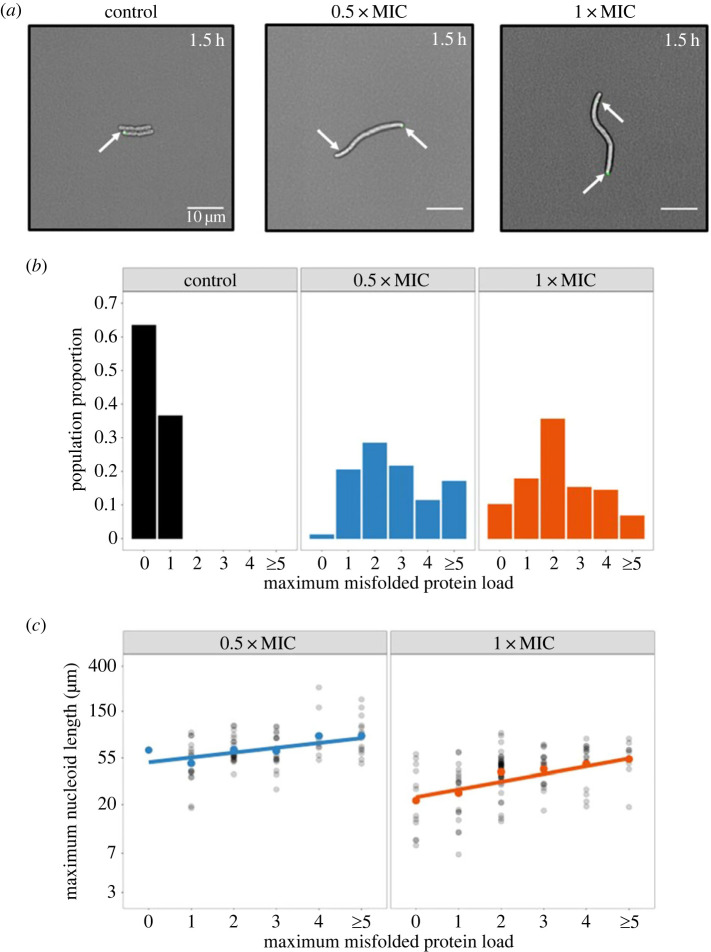
Misfolded proteins accumulate in filamentous cells. Misfolded protein load among the three treatment levels is shown. (*a*) Fluorescent phase contrast images of misfolded protein aggregation, represented by the IbpA-YFP reporter, at the two treatment levels and the control. In the control, only one of the four cells contain a misfolded protein aggregate. By contrast, at both treatment levels the filaments contain two misfolded protein aggregates, as highlighted by the white arrows. (*b*) A plot of the proportion of cells within each treatment level and their corresponding maximum misfolded protein load. The 0.5xMIC treatment has a significantly higher maximum protein load (*β* = 2.72, *n* = 310) compared to the 1xMIC treatment (*β* = 2.26, *p* = 0.036, *n* = 310) and compared to the control (*β* = 0.365, *p* < 0.1 × 10^−3^ *n* = 310). Furthermore, the 1xMIC treatment has a significantly higher maximum protein load compared to the control (*p* < 0.1 × 10^−3^ *n* = 310). (*c*) A plot of the natural log-transformed maximum nucleoid length in response to the maximum misfolded protein load at both 0.5xMIC and 1xMIC treatment levels. The centre dots signify the mean maximum nucleoid length, the straight lines represent the fitted model at each treatment level, and the black dots represent the individual data points. The maximum nucleoid length is significantly positively correlated with the maximum protein load at both the 0.5xMIC (*β* = 0.104, *p* < 0.1 × 10^−3^ *n* = 206) and the 1xMIC (*β* = 0.167, *p* < 0.1 × 10^−3^ *n* = 206) treatment level.

In addition to DNA double-strand breaks, ciprofloxacin also acts as a source of protein stress in *E. coli* [21,22]. To monitor misfolded protein aggregates by live-cell imaging in real time, we used the fluorescent construct IbpA-YFP [29]. IbpA is a bacterial heat-shock protein that binds to inclusion bodies that are aggregates of bio-macromolecules, mostly misfolded proteins. Thus IbpA serves as a detector for misfolded proteins [30,31]. We defined the maximum unfolded protein load per cell as the maximum number of IbpA-YFP foci within an individual cell across the experiment time. We fitted a Poisson mixed effects model across our data to compare the maximum protein load across the three treatment levels. Both treatment groups had significantly higher maximum IbpA-YFP foci count compared to the control, and the 0.5xMIC treatment had significantly higher maximum IbpA-YFP foci count compared to the 1xMIC treatment (figure 2*b*).

To evaluate the relationship between DNA and protein stress, we fitted a linear mixed model where the maximum nucleoid length (HU-mCherry signal) is dependent on the maximum protein load (IbpA-YFP foci) in cells under ciprofloxacin stress. We set the treatment level as a fixed effect and allowed the intercepts and slopes to vary between treatments. We found that the maximum nucleoid length was positively correlated with the maximum misfolded protein load in both the 0.5xMIC (*β* = 0.104) and 1xMIC (*β* = 0.167) treatments (figure 2*c*) and that the model explained a significant proportion of the variation in the maximum nucleoid length (marginal *R*^2^ = 0.397). The steeper slope in the 1xMIC treatment indicates that HU-mCherry nucleoid length is longer in cells with a high abundance of IbpA-YFP foci at higher levels of treatment.

### 2.3. Nucleoid elongation and a reduction in misfolded protein abundance increase survival under stress

We investigated how the nucleoid and misfolded protein dynamics of a cell relate to cell survive under ciprofloxacin treatment. We defined a binary variable that stratifies the population on whether the level of misfolded protein aggregates increases and then decreases over time (yes) or whether it increases and remains constant (no). Using a linear mixed model, we evaluated survival time as a function of the maximum HU-mCherry nucleoid length and the reduction of IbpA-YFP foci.

**Figure 3.**
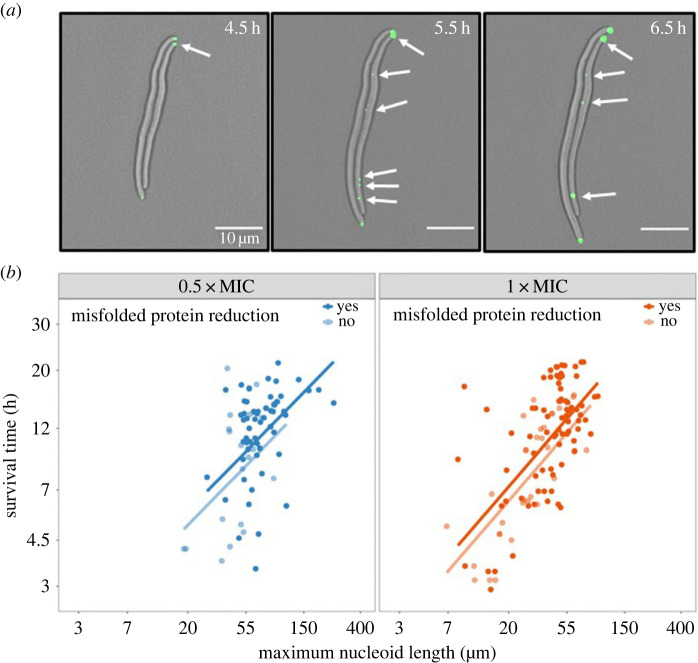
Survival time against the maximum nucleoid length stratified by a reduction in misfolded protein load. (*a*) Fluorescent phase contrast images of misfolded protein aggregation (IbpA-YFP) through time at 0.5xMIC ciprofloxacin treatment. The number of individual misfolded protein foci, highlighted by the white arrows, can be seen to decrease between hours 5.5 and 6.5 representing a reduction in misfolded protein load. (*b*) A plot of the natural log-transform survival time against the natural log-transformed maximum nucleoid length stratified by a reduction in protein load at both 0.5xMIC and 1xMIC treatment levels. The survival time is significantly positively correlated with the maximum nucleoid length at both the 0.5xMIC (*β*_1_ = 0.517, *p* < 0.1 × 10^−3^ *n* = 206) and 1xMIC (*β*_1_ = 0.588, *p* < 0.1 × 10^−3^ *n* = 206) treatment levels. Likewise, a reduction in misfolded protein load significantly increases the survival time in both the 0.5xMIC and 1xMIC treatments (*β*_2_ = 0.125, *p* = 0.022, *n* = 206).

The maximum nucleoid length was positively correlated with the survival time in both the 0.5xMIC (*β*_1_ = 0.517) and 1xMIC treatments (*β*_1_ = 0.588), regardless of reduction of misfolded protein aggregates (figure 3*b*). The steeper slope in the 1xMIC treatment suggests that an increase in nucleoid length at higher doses of ciprofloxacin results in a greater increase in survival time. Our data also showed that protein reduction was positively correlated with survival time (*β*_2_ = 0.125; figure 3*b*) suggesting that there is a survival advantage to reducing the number of misfolded proteins within a cell. The model fit was not significantly improved by estimating an independent protein reduction effect size for each treatment (*p* = 0.673). This could suggest that either the survival benefit of a reduction in misfolded protein abundance is independent of the antibiotic concentration, or that the misfolded protein dynamics at different treatment levels are more complex. The latter is possible given the stochastic events of appearance and disappearance of foci within each cell during imaging.

It is important to note that we observed a high level of variability in survival time under both 0.5xMIC and 1xMIC ciprofloxacin treatment. The model explains a greater proportion of the variation in the survival time in the 1xMIC treatment (marginal *R*^2^ = 0.460, *n* = 88) compared to the 0.5xMIC treatment (marginal *R*^2^ = 0.229, *n* = 118). This suggests that there is either a greater degree of heterogeneity in the survival time at lower treatments, or that there are additional phenotypic survival dynamics that occur at lower treatments that were not captured within the model.

### 2.4. Increased heterogeneity in misfolded protein management under lower levels of stress

**Figure 4.**
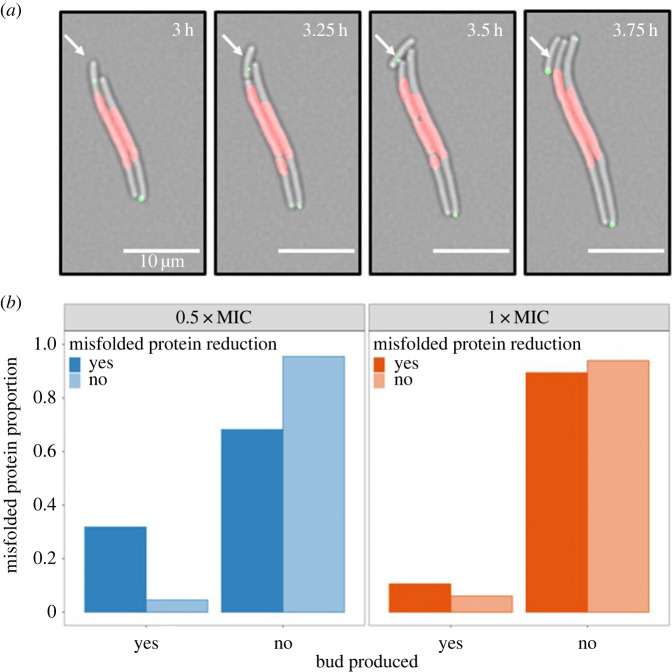
Misfolded protein buds as an alternative mechanism to reduce misfolded protein load in filamentous cells. (*a*) Fluorescent phase contrast images of misfolded protein bud production through time in response to 0.5xMIC ciprofloxacin treatment. The green is an IbpA-YFP reporter to monitor the aggregation of misfolded proteins. Aggregates of misfolded proteins are monitored in green (IbpA-YFP). Bacterial nucleoid is visualized in red (HU-mCherry). The white arrows highlight the formation of a misfolded protein bud that is void of DNA. (*b*) A plot of the proportion of cells that produce a misfolded protein bud stratified by a reduction in misfolded protein load at each treatment level. There is a significant association between a reduction in maximum misfolded protein load and the production of a misfolded protein bud in the 0.5xMIC treatment (*p* = 0.023, *n* = 88) but not in the 1xMIC treatment (*p* = 0.684, *n* = 118).

To understand the reduction in misfolded protein dynamics further, we investigated the mechanism of misfolded protein reduction in *E. coli* under treatment. Throughout the course of the single-cell tracking studies, we observed ‘budding’ of membrane-enclosed structures from the tips of filaments (figure 4*a*). While the buds frequently contained IbpA-YFP foci [32], we never observed HU-mCherry labelling, suggesting the presence of misfolded protein aggregates and the absence of chromosomal DNA.

Misfolded protein-containing buds were produced at both the 0.5xMIC and 1xMIC treatment levels. Yet, a significantly higher proportion of cells in the 0.5xMIC treatment produced a misfolded protein-containing bud compared to the 1xMIC treatment (*p* = 0.004, *n* = 206). A *χ*^2^ test showed that there is an association between a reduction in maximum misfolded protein load and the production of misfolded buds in the 0.5xMIC treatment (*p* = 0.023, *n* = 88) but not in the 1xMIC treatment (*p* = 0.684, *n* = 118; figure 4*b*). Taken as a whole, this suggests that bud production may be a mechanism to reduce the overall misfolded protein load at lower levels of treatment which is then lost under higher levels of treatment.

## 3. Discussion

We conducted phenotypic analysis into the survival dynamics of *E. coli* in response to varying levels of ciprofloxacin antibiotic treatment. Through the development of a dual reporter *E. coli* strain and a bespoke image analysis pipeline, we have generated a multidimensional dataset that captures single-cell survival dynamics through time in response to antibiotic stress. In turn, we have found that while there is a signature phenotypic stress response in *E. coli* under treatment with ciprofloxacin, the heterogeneity in the survival duration is increased at lower doses of treatment.

The first key result of this work is that increased nucleoid length promotes increased survival (figure 3). After being exposed to ciprofloxacin, a DNA-damaging drug, *E. coli* cells develop a filamentous polyploid phenotype (figures 1 and 4) with over seven times the amount of nucleoid material. Typically thought of as a driver of genomic instability and cell death [33], polyploidy can also act as a stress response mechanism and a source of evolutionary innovation [34,35] driving adaptive plasticity and resistance in response to systemic therapy [36–38]. In the present study, we show that stress-induced polyploidy acts as a survival mechanism in *E. coli* at the level of the cell (figure 3). These results are in line with previous studies which showed that multiple sets of chromosomes increase the chances of successful DNA repair via recombination [11]. Taken as a whole, these results converge on the idea that polyploidy in *E. coli*, as in other species and cancer, may act a survival strategy on a cellular level while also indirectly serving a substrate for novel evolutionary solutions, such as resistance, to emerge.

The second key result is that protein damage, as seen by the presence of IbpA-YFP foci, increases with nucleoid length, supporting the idea that protein production scales with genomic content in fast-growing bacteria (i.e. the ribosomes in excess are required to absorb the high load of protein synthesis) [39–42]. Thus, upon ciprofloxacin exposure and filament formation, an increase in genomic content is expected to correlate with an increase in protein synthesis and in the level of misfolded proteins. However, if the accumulation of protein waste exceeds the rate of protein repair or disaggregation, cellular homeostasis can be perturbed causing a halt in the DNA replication leading to unsuccessful DNA repair and ultimately, cell death [43]. This may explain why we find that there is an increased survival time in cells that reduce their misfolded protein load (figure 3). In line with this reasoning, the production of buds that clear misfolded proteins (figure 4*a*) may serve as an emergency mechanism to quickly reduce the overall misfolded protein load within the cell without the costly need for disaggregation and/or elimination by the proteasome machinery [32]. Yet, under a stress induced model of mutagenesis, the lack of bud production at higher antibiotic concentrations may suggest that budding, whilst potentially favourable, is not as closely related to fitness as a reduction in the absolute misfolded protein load. Interestingly, the emergence of such protein damage clearance phenotype in filamentous cells, may support the concept of ‘structural epistasis' recently reviewed by Baquero *et al.* [44] and may have consequences on antibiotic resistance phenotypes. Further work is needed to fully elucidate the complex misfolded protein dynamics that emerge during bacterial filamentation and specifically with regards to the effect on cellular survival and filamentation.

A pivotal next step will be to extend our analysis and quantify the phenotypic dynamics after the removal of treatment. Specifically, treatment survival does not necessarily imply successful reinitiation of division after the removal of treatment [9] that is essential for the long-term evolution of resistance. We also acknowledge that to gain a thorough understanding of the underlying evolutionary kinetics of resistance, corresponding molecular analysis in addition to the current phenotypic analysis is required. Likewise, we need to investigate the changes in dynamics over multiple generations to definitively correlate these dynamics with an increased rate of resistance. Nevertheless, we believe that this work highlights the power and importance of single-cell phenotypic analysis [9] and the need to quantify the changes in phenotypic variability as well as changes in the average phenotype.

## 4. Methods

### 4.1. Dual reporter strain construction and growth conditions

Strains used in this work are listed in the electronic supplementary material, table S2. *Escherichia coli* strains were grown at 37°C in Lysogeny broth (LB). Detailed methods for the construction of the strains *E. coli* MGAY (IbpA-YFP) and SS6279 (*hupA::hupA-mCherry*) are described in Lindner *et al.* [29] and Marceau *et al.* [45], respectively. P1 phage transduction used to move the *hupA::hupA-mCherry* (HU-mCherry) construct into SS6279 to give JB1078, was carried out as described in Miller [46]. Transductants were selected onto Kanamycin 50 µg ml^−1^ plates after one overnight of growth at 37°C and streaked out onto fresh LB plates. Transductants were imaged in the green and red channel to verify the presence of the two reporters. IbpA-YFP foci reports inclusion bodies of aggregated misfolded proteins. HupA encodes a histone-like protein decorating the DNA and thus represents a natural tracer of DNA in living bacteria. HU-mCherry fluorescence intensity reports nucleoid DNA density [47].

### 4.2. Minimum inhibitory concentration determination

The MIC, defined as the lowest concentration of ciprofloxacin that prevents any growth of the bacteria, was determined with a serial agar dilution method [48]. First, a 20 µl volume of overnight culture containing the dual reporter strain was used to inoculate 2 ml of LB medium before being placed in a shaking 37°C incubator for 2 h. Once the cells were in early log phase, a 20 µl volume was then used to inoculate 2 ml of LB medium containing increasing concentrations of ciprofloxacin (0, 10, 20, 30, 40, 50, 60 and 70 ng ml^−1^) before being placed in a shaking 37°C incubator for 24 h. The individual cultures were then serially diluted into concentrations from 1 × 10^0^ to 1 × 10^−6^ in LB medium before being spotted, in triplicate, on agar plates and then placed in a 37°C incubator for 24 h. The lowest concentration at which there was no colony growth, and thus the MIC, was 60 ng ml^−1^.

### 4.3. Imaging

In all experiments, a 20 µl volume of overnight culture was used to inoculate 2 ml of LB medium before being placed in a shaking 37°C incubator for 2 h. Once the cells were in early log phase, a 20 µl volume was then further diluted into 1 ml of LB medium to reduce the cell density during imaging. A 0.8 ml volume of cells was then transferred from the liquid culture to a 1.6% agarose-padded slide containing LB medium and either 30 or 60 ng ml^−1^ of ciprofloxacin (0.5x and 1xMIC, respectively) in the two treatment conditions. A coverslip was then placed on the pad before being sealed with valap (1 vol. vaseline/1 vol. lanolin/1 vol. paraffin) [26]. Time-lapse videos were then collected for 2 h in the control conditions and 24 h in the two treatment conditions with an image taken every 5 min. A Nikon Eclipse Ti2 Microscope (Nikon Inc.) with a live cell imaging ThermBox incubation system (Tokau Hit Co.) set at 30°C was used for all experiments. Multiple XY positions were captured during each experiment and three replicates were performed at each treatment level. To capture the images, a Nikon Plan Apo x60 oil immersion objection was used in conjunction with a Nikon DS-Qi2 camera and NIS elements version 5.11 software.

### 4.4. Image analysis

All values were quantified using a custom-built pipeline in Python [49] that can be found on GitHub (https://github.com/george-butler/bacteria_filamentation). The pipeline was designed to be highly flexible and use parallel computing where possible although high performance computing capabilities are not a necessity. The pipeline also contains several error correcting measures to try and account for unavoidable experimental deviations, e.g. changes in the focal plane over time. Furthermore, after checking we manually checked the segmented morphologies for each cell and corrected any errors.

For each cell, we quantified six phenotypic characteristics: ‘maximum filament length’, ‘maximum nucleoid length’, ‘survival time’, ‘maximum misfolded protein load’, ‘misfolded protein reduction’ and ‘bud production’:

—‘maximum filament length’ quantifies the maximum length of the filament during the timelapse video and is measured from the phase contrast channel (electronic supplementary material, figure S1);
—‘maximum nucleoid length’ quantifies the maximum length of the nucleoid within a given filament and is measured from the red fluorescent channel (electronic supplementary material, figure S1). Owing to experimental limitations, the tracking of a given cell in the phase contrast channel may stop prior to the tracking of the same cell in the red fluorescent channel. As a result, the maximum nucleoid length in a given cell may be longer than the maximum filament length in the same cell. To ensure that loss of focus was not treatment specific, and thus not potentially biasing our results, we used a *χ*^2^ test to evaluate whether there was an association between the antibiotic concentration and the proportion of cells in which the nucleoid length was longer than the filament length. We found that there was no significant difference in the proportion of cells that had a longer nucleoid length compared to their filament length in the two treatment conditions;
—‘survival time’ was recorded as the total time that cell was alive. That is, the time between the start of tracking until the death of the filament as characterized by a loss in HupA signal;
—‘maximum misfolded protein load’ was calculated as the maximum number of IbpA foci recorded within a cell prior death (figure 2*a*). Owing to a small number of cells having an extremely large number of foci we clustered all cells that had between 5 and 9 IbpA foci as greater than or equal to 5;
—‘misfolded protein reduction’ is a binary variable that records whether the number of individual protein foci within a cell decreases after the maximum protein load has been reached (figure 3*a*); and
—‘bud production’ is a binary variable that records whether a protein aggregate has been disposed of in a ‘bud’ by tracking each IbpA foci within a cell (figure 4*a*). That is, after tracking, each IbpA foci is then allocated a ‘bud probability’ that represents the likelihood that the foci has been removed from the cell in a bud versus the signal being lost owing to degradation of the protein aggregate. A threshold is then set to identify cells that have disposed of a misfolded protein in a bud. A 95% threshold was used throughout this analysis although the same qualitative results were also achieved with a 90% threshold.

### 4.5. Statistical analysis

All statistical analysis was performed in R [50], and figures were made using ggplot [51]. All code and corresponding data can be found on GitHub (https://github.com/george-butler/bacteria_filamentation). To be included in the final dataset, a cell needed to be present for at least 50% of the tracked time course. Some cells were not detected in a given frame or had to be removed owing to segmentation inaccuracies. In the two treatment conditions, we also excluded cells that were initially present at the start of the video. Instead, we only considered cases in which a cell division had been observed and then recorded the dynamics of the progeny to ensure that survival time was as accurate as possible. Finally, in the two treatment conditions, each progeny had to have died during the 24 h time course to be included in our phenotypic analysis. This criterion is essential to ensure that we captured the full life cycle of each cell in response to treatment in our analysis.

Throughout our analysis, we used linear mixed models to account for the differences between replicate populations and different imaging locations [52], except for in figure 4. The maximum nucleoid length against the maximum filament length (figure 1) was estimated with a single treatment intercept and independent slopes for each treatment. The single intercept is important because populations in each experiment are derived from the same ancestor population. As a result, the average survival prior to the application of treatment, and thus prior to nucleoid elongation, is expected to be derived from the same underlying distribution. The maximum nucleoid length against the maximum protein load was estimated with independent intercepts and slopes for each treatment (figure 2). The survival time against the maximum nucleoid length and the reduction in maximum protein was estimated with a single treatment intercept and independent slopes for each treatment (figure 3). Each model was selected through a process of forward selection whereby parameters were only included if they were significant at the 5% level. The marginal *R*^2^ values were calculated using the methods detailed by Nakagawa & Schielzeth [53].

## Data Availability

Data and relevant code for this research work are stored in GitHub: https://github.com/george-butler/bacteria_filamentation and have been archived within the Zenodo repository [54]: https://doi.org/10.5281/zenodo.8090711. Raw image data are available from George Butler (gbutle16@jh.edu). Processed data are available on GitHub (https://github.com/george-butler/bacteria_filamentation), and all raw image data is available from George Butler (gbutle16@jh.edu). The data are provided in the electronic supplementary material [55].
